# Cue-induced inhibitory control in forensic patients with alcohol use disorder: A link to criminal recidivism risk assessed by factor 2 psychopathy

**DOI:** 10.1016/j.abrep.2026.100713

**Published:** 2026-05-27

**Authors:** Helena Sophia Schmitt, Jennifer Wernicke, Cornelia Sindermann, Ralf Wilhelm Wolf, Sebastian Markett, Esther Ott, Christian Montag

**Affiliations:** a Department of Personality Psychology, Institute of Psychology and Education, Ulm University, Ulm, Germany; b Department of Forensic Psychiatry and Psychotherapy, Ulm University, Ulm, Germany; c Psychological Assessment, Differential Psychology, and Psychological Methods, Charlotte Fresenius Hochschule - University of Psychology, Heidelberg, Germany; d Computational Digital Psychology, Interchange Forum for Reflecting on Intelligent Systems, University of Stuttgart, Stuttgart, Germany; e Vitos Clinic for Forensic Psychiatry Hadamar, Hadamar, Germany; f Department of Psychology, Humboldt-Universität zu Berlin, Berlin, Germany; g Centre for Cognitive and Brain Sciences, Institute of Collaborative Innovation, University of Macau, Macau SAR, China; h Department of Psychology, Faculty of Social Sciences, University of Macau, Macau SAR, China; i Department of Computer and Information Science, Faculty of Science and Technology, University of Macau, Macau SAR, China

**Keywords:** Inhibitory control, Cue-reactivity, Reoffending, Risk assessment, Forensic psychiatry, Drug-crime link

## Abstract

Cue-reactivity and impaired inhibitory control represent well-established mechanisms contributing to substance use relapse, which, in turn, increases the risk of criminal reoffending in forensic populations whose offending behavior is closely linked to substance use. In Germany, such individuals may be mandated to custodial addiction treatment under Section 64 of the Criminal Code (§ 64 StGB). To date, it remains largely unexplored whether inhibitory control deficits under substance-cue exposure relate more directly to indicators of criminal recidivism risk in forensic addiction populations. The present work addresses this gap by examining the relationship between cue-induced inhibitory control and a validated proxy of reoffending risk.

Fifty-one abstinent male forensic patients (24 with alcohol use disorder (AUD), 27 with other substance use disorders (SUD)) undergoing treatment under Section 64 completed two Go/NoGo tasks assessing inhibitory control under alcohol-related and neutral stimulus conditions. Controlling for relevant covariates, inhibitory performance (adjusted $d^{\prime }$) was used to predict scores on Factor 2 of the Psychopathy Checklist: Screening Version (PCL:SV).

Among patients with AUD, weaker inhibitory control in the alcohol-cued condition was strongly associated with higher PCL:SV Factor 2 scores (β = −0.79, *p* = .004), whereas no such association emerged for the neutral condition or for patients with other SUDs. These findings suggest a context-specific link between cue-elicited inhibitory deficits and criminogenic risk in forensic patients with AUD.

Cue-related inhibitory impairments may represent a key cognitive mechanism connecting substance use to criminal (re-)engagement. If replicated longitudinally, such measures may inform future approaches to risk assessment and intervention in forensic addiction treatment.

## Highlights

• Alcohol-cue inhibitory deficits predict criminogenic risk in forensic patients with alcohol use disorders.
• Neutral vs. alcohol-cue Go/NoGo task linked to reoffending risk measure.
• No alcohol-cue effect observed in forensic patients with other substance use disorders.
• Cue-elicited disinhibition may connect substance relapse mechanisms and crime.
• Findings inform cue-focused risk assessment and intervention strategies.

## 1 Introduction

Forensic addiction treatment under Section 64 of the German Criminal Code (Strafgesetzbuch; StGB) provides the legal framework for the court-ordered placement of individuals with substance use problems who have committed criminal offenses in the context of substance use. Such placement presupposes that treatment in a specialized withdrawal facility is expected to reduce future substance-related offending by alleviating the underlying disorder or preventing relapse over a substantial period. Accordingly, Section 64 pursues a dual objective: Protecting the public while offering therapeutic intervention targeting criminogenic substance use. The effectiveness of forensic addiction treatment is therefore commonly evaluated not only in terms of clinical outcomes, but also with respect to criminal recidivism following discharge (Müller et al., 2017).

Despite sustained legal debate and a recent reform that tightened causal and prognostic thresholds for placement (Schwarz & Stübner, 2023; Soyka, 2024), outcome-oriented catamnestic research consistently demonstrates that successful completion of forensic addiction treatment under Section 64 is associated with substantially reduced criminal recidivism compared to imprisonment or unsuccessful treatment trajectories (i.e., premature discontinuation). Mounting evidence suggests that patients released regularly after treatment completion show markedly lower reoffending rates and a delayed onset of reoffending relative to individuals released from prison or those whose treatment was prematurely terminated (Dudeck et al., 2018; Querengässer et al., 2018, Querengässer et al., 2025; Schalast et al., 2021). Empirical estimates illustrate the magnitude of this benefit, with one-year recidivism rates among successfully treated individuals typically ranging between approximately 17% and 29%, whereas corresponding rates in non-treatment groups or prematurely discharged patients often exceed 40% and reach beyond 50% (Querengässer et al., 2018, Querengässer et al., 2025; Schalast et al., 2021).

At the same time, longer follow-up periods demonstrate that placement in withdrawal centers does not confer absolute protection against criminal relapse. Even among successfully treated individuals, the risk of reoffending increases over time, with approximately half acquiring a new entry in the Federal Central Criminal Register within three years after discharge (Querengässer et al., 2018; Schalast et al., 2021). This persistence of risk underscores the need to better understand the mechanisms that contribute to criminal relapse after treatment completion.

Relapse into substance use represents a central pathway to criminal recidivism following forensic addiction treatment, with reoffending occurring predominantly among individuals who resume substance use after discharge (Bezzel, 2010; Passow et al., 2015; Pfaff, 1998). This consistent pattern underscores the criminogenic role of substance use and highlights abstinence maintenance and relapse prevention as core targets of forensic treatment and aftercare. Consequently, identifying mechanisms that increase vulnerability to substance use relapse may also provide critical insight into pathways leading to renewed criminal behavior.

One such mechanism is cue-reactivity, defined as conditioned motivational, affective and physiological response to substance-related stimuli. Decades of experimental and clinical research have demonstrated that individuals with substance use problems exhibit elevated reactivity to such cues, which may manifest in subjective (e.g., craving, stress, arousal), neurological, physiological (e.g., skin conductance, salivation) and/or behavioral (e.g., substance-seeking) responses, and ultimately trigger substance use and relapse (Carter & Tiffany, 1999; Sinha & Li, 2007). Importantly, a recent large-scale meta-analysis demonstrates that cue-reactivity shows robust prospective associations with subsequent substance use and relapse, with cue- and craving-related responses more than doubling the odds of future use across a wide range of substances (Vafaie & Kober, 2022). From a neurobehavioral perspective, cue-reactivity arises from associative learning processes through which substance-related stimuli acquire motivational significance and begin to bias behavior. With repeated pairing, these cues become increasingly salient and capable of eliciting craving and approach tendencies, even in the absence of deliberate intention. This heightened cue sensitivity is thought to reflect dopaminergic sensitization, whereby drug associated stimuli come to be disproportionately “wanted” and exert strong bottom-up control over behavior (Robinson and Berridge, 1993, Robinson and Berridge, 2025). At the same time, weakened top-down inhibitory control limits the ability to suppress cue-triggered responses, together creating a state of elevated relapse vulnerability in which substance-seeking behavior can readily re-emerge (Goldstein and Volkow, 2002, Goldstein and Volkow, 2011).

These theoretical accounts are of particular interest for forensic contexts, where individuals are expected to maintain substance abstinence and lawful behavior despite potential re-exposure to environments rich in substance-related cues. Such cue exposure can challenge self-regulation, particularly among individuals with impaired executive functioning, including inhibitory control, a deficit frequently observed in both substance use disorders and offending populations (Ogilvie et al., 2011; Smith et al., 2014; Vedelago et al., 2019). Compromised inhibitory control may reduce the ability to resist cue-triggered impulses, thereby increasing vulnerability for substance use relapse and, in turn, elevating the risk of renewed criminal behavior.

Supporting this view, converging evidence indicates that impaired inhibitory control and heightened cue-reactivity frequently co-occur. Individuals with heavier drinking patterns exhibit weaker response inhibition and stronger cue-induced craving in the presence of alcohol-related stimuli, whereas such effects are markedly less pronounced in light drinkers (Ames et al., 2014; Papachristou et al., 2012, Papachristou et al., 2013). Moreover, exposure to alcohol cues has been shown to disrupt inhibitory control and amplify craving, with both processes contributing to increased alcohol consumption under laboratory conditions (Field & Jones, 2017). Together, these findings suggest that substance-related cues may reveal and exacerbate inhibitory control difficulties, jointly elevating vulnerability to substance use relapse.

### 1.1 The present work

Despite substantial evidence linking cue-reactivity and impaired response inhibition to substance use, it remains largely unexplored whether deficits in inhibitory control under cue-exposure conditions are also associated with criminal recidivism risk in forensic patients.

The present study addresses this gap by linking laboratory indices of cue-elicited inhibitory control with a validated forensic risk measure in patients committed under Section 64 of the German Criminal Code. Specifically, inhibitory control is assessed under neutral and alcohol-cue exposure conditions in forensic patients with an alcohol use disorder (AUD) and in those with other substance use disorders (SUD). Inhibitory performance is studied in relation to Factor 2 (Lifestyle/Antisocial) scores of the Psychopathy Checklist: Screening Version (PCL:SV, Hart et al. (1995)), a well-established indicator of criminogenic risk with robust criterion validity for general and violent recidivism across meta-analytic evidence (DeMatteo & Olver, 2022; Holper et al., 2025; Yang et al., 2010).

Accordingly, the present study examines whether variation in inhibitory control under different cue conditions is associated with an established proxy of criminogenic risk, while controlling for general executive functioning using a working memory measure. By integrating experimentally manipulated cue-exposure paradigms with this risk framework, the study adopts a novel approach to examining substance- and context-specific links between inhibitory control and criminogenic risk across diagnostic groups.

### 1.2 Hypotheses

It is hypothesized that weaker inhibitory control will be associated with higher PCL:SV Factor 2 scores, reflecting increased criminogenic risk. This association is expected to be condition- and diagnosis-specific. In patients with AUD, inhibitory control impairments under alcohol-cue exposure are hypothesized to show the strongest association with reoffending risk, given the heightened motivational salience of alcohol-related cues in this group. Patients with other SUDs serve as a diagnostic comparison group, with associations examined exploratorily.

In addition, cue-reactivity (i.e., arousal and craving responses) is examined in relation to inhibitory control. It is expected that stronger cue-related responses will be associated with reduced inhibitory control, particularly under alcohol-cue exposure in the AUD group. Given the primary focus of the present work on the relationship between inhibitory control under cue-exposure and forensic risk assessment, these analyses are considered secondary in nature.

## 2 Materials and methods

### 2.1 Procedure

Data were collected in a high-security forensic psychiatric facility (Maßregelvollzug) in the federal state of Hesse, Germany, involving patients committed under Section 64 of the German Criminal Code. Recruitment and assessment took place in three waves between April 2019 and November 2022. All data were collected prior to the reform of Section 64, resulting in a legally homogeneous sample. The study employed a cross-sectional design within a larger research project assessing additional personality and behavioral measures not relevant to the present analyses. Eligibility criteria included legal adulthood (i.e., at least 18 years of age), commitment under Section 64, sufficient German language proficiency, and the ability to provide informed consent. Exclusion criteria were documented intellectual disability or impaired capacity to consent.

**Fig. 1:**
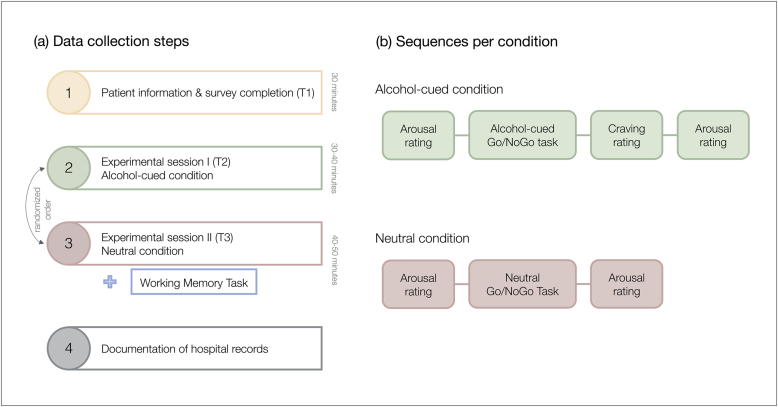
**(a)** Data collection steps and **(b)** procedure in each condition of the Go/NoGo task. *Notes.* Shown is an exemplary study timeline for a participant who completed the alcohol-cued condition of the Go/NoGo task at T2 and the neutral condition at T3. Assignment to the alcohol-cued and neutral conditions at T2 and T3 was randomized across participants; accordingly, the order in which the two conditions were administered varied. At T2 and T3, participants always completed opposite conditions. The mean interval between the two experimental sessions (T2 and T3) was, on average, *M* = 5.21 (*SD* = 2.65) days. The depicted session duration refers to the total length of each appointment and therefore also includes assessment instruments and behavioral measures that were not part of the present investigation.

Data collection comprised three appointments per participant (see Fig. 1a). At the first appointment (T1), participants received study information, provided written informed consent, and completed self-report questionnaires. The second (T2) and third (T3) appointments involved experimental testing sessions. Participants completed two visual Go/NoGo tasks assessing inhibitory control under alcohol-cue and neutral conditions (see Fig. 1b), with condition order randomized across participants. The mean interval between T2 and T3 was *M* = 5.21 days (*SD* = 2.65). At each experimental session, current arousal was assessed before and after the task to capture general state changes. Craving ratings were obtained only following the alcohol-cue condition to avoid potential priming effects associated with explicit pre-task assessment of alcohol-related motivational states.

A working memory task conducted at the end of T3 was included to account for individual differences in general executive functioning in the present analyses.

Total participation time across all sessions was approximately 1.5 to 2 h. Participants received financial compensation upon completion.

Following completion of all assessments, relevant clinical file information (e.g., offense characteristics, diagnoses, and PCL:SV data) was extracted in collaboration with clinical staff.

Data were linked using pseudonymous codes and subsequently anonymized. All participants provided written informed consent. Ethical approval was obtained from the Ethics Committee of the State Medical Association of Hesse (FF17/2018) and the Ethics Committee of Ulm University (337/17).

### 2.2 Sample

Data were initially collected from 81 patients. For the present research question, analyses were restricted to a subsample defined by a priori inclusion criteria focusing on male patients with complete datasets for all variables of interest. Restriction to male participants was applied due to documented gender differences in psychopathy assessments and their criterion validity (De Vogel & Lancel, 2016; Holper et al., 2025; Klein Haneveld et al., 2022), as well as the small number of women with AUD (*n* = 5), which precluded meaningful diagnostic subgroup analyses.

Of the remaining 64 male patients, two cases with combined measures under Sections 63 and 64 of the Criminal Code were excluded due to their distinct clinical and legal characteristics.

Participants without an available PCL:SV assessment (*n* = 4) or with missing data in the inhibitory control (*n* = 3) or working memory *(n* = 2) tasks were also excluded. Missingness was attributable to administrative timing (i.e., incomplete PCL assessments) and technical issues during data collection rather than patient characteristics, suggesting that data were unlikely to be systematically missing. Given the small number of affected cases, a complete-case approach was applied.

After additionally removing *n* = 2 cases due to outlying data in the inhibitory control tasks (see **Supplementary Materials S1**), the final analytic sample consisted of *N* = 51 individuals, of whom *n* = 24 met diagnostic criteria for AUD and *n* = 27 were diagnosed with other SUDs.

Descriptive statistics for the sample are presented in Table 1. Offense categories refer to the primary index offense; where multiple offenses were present, the most severe offense is reported.

**Table 1:** Sample characteristics of the full sample (*N* = 51) alongside comparisons between the diagnostic groups with AUD and other SUDs.

**Continuous variables**		***M***	***SD***	***t (df)***	***p***	***Cohen's d***
Age		34.82	7.96	2.06 (49)	0.044	0.579
Sentence (in months)		52.33	23.08	0.05 (49)	0.962	0.014
Duration of admission (in days)		418.76	222.39	0.11 (49)	0.913	0.031

**Categorical variables**		***n***	***%***	***Likelihood Ratio***χ**^2^*****(df)***	***p***	***Cramer's V***

Formal Education (initial six categories)				10.13 (5)	0.072	0.400
	None	6	11.76			
	Secondary school leaving certificate (graduation after 9 years)	23	45.10			
	Intermediate school leaving certificate (graduation after 10 years)	17	33.33			
	Vocational diploma	3	5.88			
	High school diploma	1	1.96			
	College degree	1	1.96			
Education (aggregated three categories)				5.64 (2)	0.059	0.330
	No degree	6	11.76			
	Lower secondary degree	23	45.10			
	Intermediate or higher degree	22	43.14			
Marital status				3.75 (3)	0.290	0.242
	Single	36	70.59			
	Married/registered civil partnership	8	15.69			
	Divorced	5	9.80			
	Separated	2	3.92			
Nationality^1^				1.04 (1)	0.308	0.143
	German	38	74.51			
	Other	12	23.53			
Diagnosis (ICD-10)						
	*Mental and behavioral disorders due to the use of*^2^					
	Alcohol (F10.x)	24	47.06			
	Opioids (F11.x)	5	9.80			
	Cannabinoids (F12.x)	26	50.98			
	Sedatives (F13.x)	1	1.96			
	Cocaine (F14.x)	21	41.18			
	Psychostimulants (F15.x)	9	17.65			
	Hallucinogens (F16.x)	1	1.96			
	Multiple drug use (F19.x)	15	29.41			
	*Personality disorders*^3^			10.58 (7)	0.158	0.393
	Dissocial (F60.2)	6	11.76			
	Emotionally unstable, impulsive type (F60.30)	1	1.96			
	Emotionally unstable, borderline type (F60.31)	3	5.88			
	Anxious (avoidant) (F60.6)	1	1.96			
	Narcissistic (F60.81)	1	1.96			
	Other specific personality disorders (F60.89)	1	1.96			
	Mixed and other personality disorders (F61)	3	5.88			
	*Other*^3^			4.88 (4)	0.300	0.270
	Schizoaffective disorder, mixed type (F25.2)	1	1.96			
	Pathological gambling (F63.0)	3	5.88			
	Socialized conduct disorder (F91.2)	1	1.96			
	Other conduct disorders (F91.8)	1	1.96			
Main index offense				16.22 (10)	0.093	0.503
	Attempted murder	1	1.96			
	Attempted homicide	2	3.92			
	Assault	6	11.76			
	Sexual assault	1	1.96			
	Robbery	17	33.33			
	Property crime (Burglary/Theft)	8	15.69			
	Drug offenses (Violation of the Narcotic Drugs Act)	12	23.53			
	Arson	1	1.96			
	Fraud	1	1.96			
	Forgery	1	1.96			
	Traffic offense	1	1.96			

**Dichotomous variables**		***n***	***%***	***Fisher's exact p***		***Cramer's V***

Smoker		44	86.27	1.000		0.034
Psychotropic medication intake (regular/as needed)		17	33.33	1.000		0.000
	Antidepressants	7	13.73	1.000		0.034
	Antipsychotics/Neuroleptics	10	19.61	1.000		0.029
	Mood stabilizers	3	5.88	0.097		0.265
	Sedative/Hypnotic agents	3	5.88	0.097		0.265

*Notes*. Continuous variables were compared using independent-samples *t-*tests. Categorical variables were analyzed using likelihood ratio χ^2^ tests or Fisher's exact tests, as appropriate. Effect sizes are reported as Cohen's *d* and Cramer's *V*. All tests were two-tailed. *M* = Mean, *SD* = Standard Deviation, *t* = t-test statistic, *df* = degrees of freedom.

1 Missing information from *n* = 1 case.

2 Because some participants had more than one substance use diagnosis, multiple entries occur and the frequencies therefore exceed 100%.

3 Because only a few participants had been diagnosed with comorbid disorders, the reported frequencies do not add up to 100%.

### 2.3 Measures

All computerized behavioral tasks were programmed in E-Prime 2.0 Professional (Psychology Software Tools, Inc., 2012) and administered on a standard 15-inch laptop computer in a supervised setting.

#### 2.3.1 Psychopathy checklist: Screening version

PCL:SV (Hart et al., 1995) data were obtained from clinical records. Assessments are routinely conducted by trained therapists in the facility. When multiple assessments were available, the most recent was used.

The PCL:SV comprises 12 items that assess core psychopathic facets based on file information and semi-structured interviews. Ratings are made on a three-point scale (0 = not present, 1 = possibly present, 2 = definitively present), yielding a total score ranging from 0 to 24. Each facet is represented by three items, and the four facets can be aggregated into two higher-order factors: *Interpersonal/Affective* (Factor 1) and *Lifestyle/Antisocial* (Factor 2). Given its established predictive validity for recidivism (DeMatteo & Olver, 2022; Holper et al., 2025; Yang et al., 2010), Factor 2 was selected as the primary outcome measure. Factor 2 comprises impulsivity, poor behavioral control, and the lack of realistic long-term goals (together constituting the *Lifestyle* facet), as well as irresponsibility and antisocial behavior in adolescence and adulthood (together constituting the *Antisocial* facet). Based on these six items, scores between 0 and 12 can be obtained for PCL:SV Factor 2. Internal consistency in the present sample was acceptable (α = 0.713, ω = 0.688).

#### 2.3.2 Go/NoGo task

Inhibitory control under alcohol-related versus neutral conditions was operationalized using visual Go/NoGo tasks. In both conditions, participants were required to execute rapid responses to frequent Go stimuli while withholding responses to infrequent NoGo stimuli, thereby indexing the ability to inhibit prepotent motor responses (Diamond, 2013). The general task structure was identical across conditions, with the stimulus content differing between them.

**Fig. 2:**
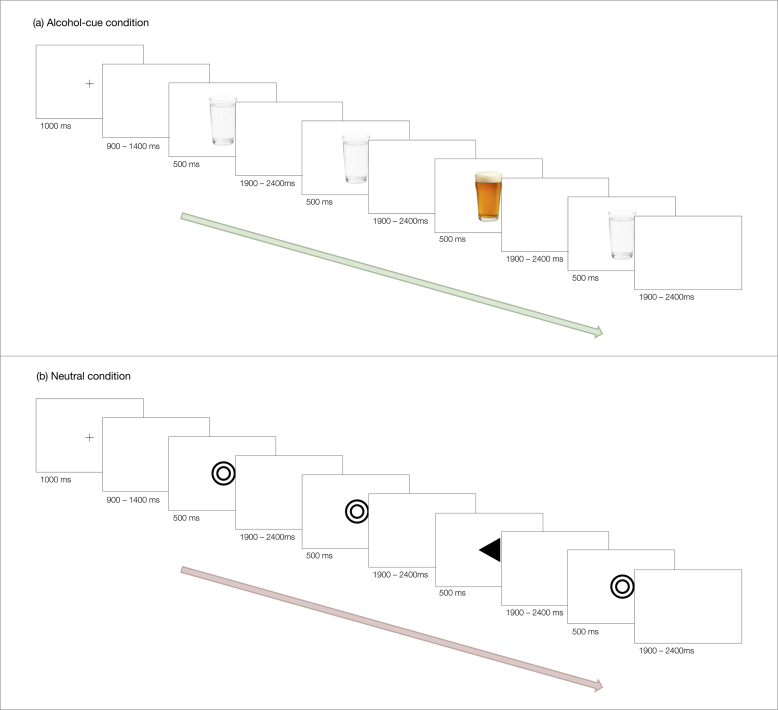
Trial structure of the Go/NoGo task in the **(a)** alcohol-cue exposure and **(b)** neutral conditions. *Notes.* Displayed are exemplary blocks of each condition. The temporal structure and task parameters were identical across conditions; only stimulus content differed. Each condition comprised six blocks. Each block began with a central fixation cross (1000 ms). Trials started with a blank intertrial interval (ITI; randomized 900–1400 ms), followed by stimulus presentation (500 ms). A subsequent blank screen served as the response window (1000 ms) and continued into the next ITI, resulting in a total stimulus-to-stimulus interval of 1900–2400 ms. Each block comprised 80 trials with a Go:NoGo ratio of 3:1. ms = milliseconds.

In the alcohol-cue condition, stimuli consisted of colored images of alcoholic and non-alcoholic beverages (Ames et al., 2014; Kreusch et al., 2014), with non-alcoholic drinks serving as Go stimuli and alcoholic drinks (beer, vodka, and whiskey) as NoGo stimuli. In the neutral condition, simple geometric shapes were presented (Benikos et al., 2013). Condition order was randomized across participants. The temporal structure of the task is illustrated in Fig. 2.

Task performance was quantified using the sensitivity parameter d′ from signal detection theory, reflecting the difference between hit rates and false alarm rates (Macmillan, 2002). A log-linear correction was applied to account for extreme proportions (Hautus, 1995), yielding the adjusted sensitivity metric $d\prime_{\mathrm{adj}}$. Mean $d\prime_{\mathrm{adj}}$ values were computed across the six blocks for the alcohol and neutral conditions. Both conditions demonstrated good internal consistency (neutral: α = 0.857, ω = 0.859; alcohol: α = 0.849, ω = 0.852).

Detailed descriptions of task parameters, stimuli, and preprocessing procedures are provided in **Supplementary Materials S1.**

#### 2.3.3 Cue-reactivity measures

Cue-reactivity was assessed using subjective arousal and craving ratings, representing established indicators of conditioned responses to substance-related cues (Carter et al., 2009; Carter & Tiffany, 1999).

##### 2.3.3.1 Arousal ratings

Before and after each inhibitory control task, participants reported their current level of arousal. Arousal was assessed using the Self-Assessment Manikin (SAM; Bradley and Lang (1994)), a nonverbal, image-based 9-point rating scale (1 = calm to 9 = excited). For all analyses, post-task arousal ratings were examined while statistically controlling for pre-task (baseline) arousal levels.

##### 2.3.3.2 Craving assessment

Following completion of the alcohol Go/NoGo task, participants' current craving was assessed. In line with the Ambivalence Model of Craving (Schlauch et al., 2015), both approach (desire to drink) and avoidance (desire to refrain from drinking) craving were measured. Participants indicated their desire to drink and their desire to not drink each alcoholic beverage (beer, vodka, and whiskey) on a 9-point rating scale (1 = not at all to 9 = very much). Mean scores for approach craving and avoidance craving were computed.

Due to a technical error in the first data collection wave, craving data were available for a reduced subsample (*n* = 33). Two participants showing zero intra-individual variance across items were excluded, resulting in a final subsample of *n* = 31 (*n*_*AUD*_ = 12; *n*_*SUD*_ = 19) for craving analyses. Internal consistency of the approach craving scale improved after removal of one item (final α = 0.625). The avoidance craving scale showed low internal consistency (α = 0.581; ω = 0.582), which may reflect the small number of items.

#### 2.3.4 Working memory task

Working memory capacity was included to account for individual differences in general executive functioning and to isolate inhibitory control effects. Performance in Go/NoGo paradigms depends not only on response inhibition, but also on the ability to maintain task rules and monitor infrequent events, which places demands on working memory (Diamond, 2013; Friedman & Miyake, 2017). Accordingly, working memory was included as a control variable.

**Fig. 3:**
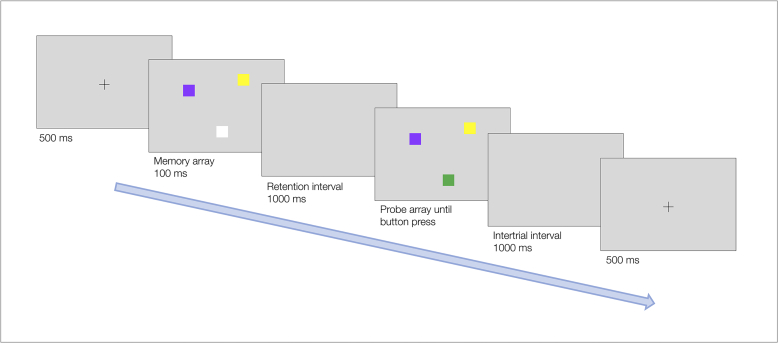
Experimental setup of the working memory task. *Notes.* Displayed is an exemplary change trial with three items. In change trials, the memory and probe arrays differed in the color of exactly one square. In catch trials, the memory and probe arrays were identical. The task comprised a total of four blocks, with each block consisting of 32 trials (16 catch and 16 change trials). The number of squares increased by one from block to block, from three squares in Block 1 to six squares in Block 4. ms = milliseconds.

Working memory capacity was assessed using a change-detection task following Luck and Vogel (1997) and Markett et al. (2018). Participants completed four blocks of trials with increasing memory load (see Fig. 3). Individual capacity was calculated for each block using the Pashler formula (Pashler, 1988), and the highest K value across blocks was used as the index of working memory capacity.

Detailed task procedures, stimulus parameters, and preprocessing steps are provided in **Supplementary Materials S2**.

#### 2.3.5 Covariates

Age, educational attainment, and psychotropic medication intake were considered as potential covariates due to their relevance for criminal behavior and/or cognitive performance (Baliousis et al., 2019; Baune et al., 2018; Chandramouleeshwaran et al., 2024; Cortés Pascual et al., 2019; Ferguson et al., 2021; Haime et al., 2021; Keefe, 2007; Liu et al., 2020; Olver & Wong, 2015; Prado et al., 2018).

### 2.4 Data analytic plan

Patient data that were available only in paper form (including clinical record data) were digitized and independently verified prior to analysis. Preprocessing of behavioral task data was conducted in RStudio version 1.3.1073 “Giant Goldenrod” (RStudio Team, 2020) using R version 4.0.2 “Taking Off Again” (R Core Team, 2020), and statistical analyses were performed in IBM Statistics Program for the Social Sciences (SPSS) version 31.0 (IBM Corp., 2025).

Percentile-based winsorization (5th/95th percentile) was applied to key study variables (PCL:SV Factor 2, inhibitory control $d\prime_{\mathrm{adj}}$ in both conditions, working memory capacity K, pre- and post-task arousal, approach and avoidance craving) within diagnostic groups to limit the influence of single extreme observations and to increase the robustness of the regression estimates.

Group comparability (AUD vs. SUD) was examined with regard to relevant sociodemographic, clinical, and offense-related characteristics, using appropriate tests for continuous and categorical variables (please see Table 1). Due to sparse cell counts, the original six-level education variable was recoded into a three-level ordinal variable (no degree, lower secondary degree, intermediate or higher degree) for all subsequent analyses.

Due to non-normal distributions, nonparametric tests were used for group comparisons of key study variables (see Table 2).

**Table 2:** Descriptive statistics for the full sample (*N* = 51).

	*M*	*SD*	*Min*	*Max*	*Median*	*Shapiro-Wilk*		*Mann-Whitney*	
						*W*	*p*	*U*	*p*
Factor 2 Psychopathy	6.84	2.86	1.30	11.00	7.00	0.946	0.021	367.50	0.409
Inhibitory control alcohol condition	2.74	0.41	1.97	3.33	2.81	0.935	0.008	343.00	0.720
Inhibitory control neutral condition	2.85	0.42	1.96	3.45	2.90	0.932	0.006	322.00	0.970
Working memory capacity	3.84	0.76	2.34	5.24	3.73	0.957	0.061	344.50	0.699
Approach craving^a^	1.78	1.43	1.00	5.65	1.00	0.615	<0.001	117.00	0.921
Avoidance craving^a^	7.67	1.78	3.67	9.00	8.67	0.750	<0.001	94.50	0.435
Pre-task arousal alcohol condition	5.03	1.82	2.00	8.00	5.00	0.935	0.008	359.00	0.503
Post-task arousal alcohol condition	5.18	1.68	2.00	7.85	5.00	0.935	0.008	368.50	0.394
Pre-task arousal neutral condition	4.57	1.69	1.30	7.70	5.00	0.956	0.059	354.00	0.566
Post-task arousal neutral condition	4.85	1.94	1.30	8.00	5.00	0.937	0.009	368.50	0.394

*Notes. M* = Mean, *SD* = Standard Deviation, *Min* = Minimum, *Max* = Maximum, *W* = Shapiro-Wilk test statistic, *U* = Mann-Whitney *U* test statistic, Inhibitory control refers to $d\prime_{\mathrm{adj}}$ scores derived from the Go/NoGo tasks; Working memory capacity refers to parameter K of the working memory task. All *p-*values are based on two-sided tests.

a Descriptive statistics are calculated based on a smaller sample of *n* = 31 (please see *Craving assessment* in the ***Measures*** section).

Potential covariates (age, education, psychotropic medication intake) were evaluated using Spearman's rank correlation coefficient ρ and included in regression models if significantly associated with the outcome or predictor variables (*p* < .05).

Secondary analyses tested associations between inhibitory control and cue-reactivity indices, i.e., post-task arousal (controlling for baseline) as well as approach and avoidance craving. Associations between inhibitory control and post-task arousal in the neutral condition served as a reference. To test the primary hypothesis, linear regression models were used to assess whether inhibitory control under alcohol-cue and neutral conditions predicted PCL:SV Factor 2 scores, while controlling for working memory capacity. Due to the diagnosis-specific nature of the research question, analyses were conducted separately for each diagnostic group.

Regression assumptions were evaluated and found to be met. Sensitivity analyses indicated that results were robust to potential outliers; therefore, all cases were retained. Ordinary least squares (OLS) regression models were conducted in both groups. Detailed regression diagnostics are provided in **Supplementary Materials S3**.

## 3 Results

Sample characteristics are presented in Table 1. Overall, the AUD and SUD groups did not differ across most sociodemographic, clinical and offense-related variables. A significant group difference emerged for age, with patients in the SUD group being slightly older on average (*p* = .044). Education showed a marginal difference in distribution between groups (*p* = .059).

Table 2 presents the descriptive statistics for all study variables for the full sample, including tests of normality and group comparisons. No significant group differences were observed for the primary study variables.

Bivariate associations among the key study variables and covariates (age, education, and psychotropic medication intake) are reported separately for the SUD and AUD samples in Table 3, Table 4. As noted in the *Measures* section, correlations involving craving variables are based on reduced sample sizes.

**Table 3:** Bivariate Spearman rank correlation coefficients (ρ) in the SUD sample (*n* = 27).

		**1**	**2**	**3**	**4**	**5**	**6**	**7**	**8**^a^	**9**^a^	**10**^b^
**1**	Age										
**2**	Education	0.42*									
**3**	Psychotropic medication intake	0.32	0.35								
**4**	Factor 2 Psychopathy	−0.50**	−0.26	0.02							
**5**	Inhibitory control alcohol condition	0.30	0.59**	0.02	−0.14						
**6**	Inhibitory control neutral condition	0.50**	0.49**	0.13	−0.41*	0.67***					
**7**	Working memory capacity	−0.23	0.09	0.08	0.27	−0.14	0.02				
**8**	Approach craving^a^	−0.06	0.19	0.07	−0.03	0.21	−0.06	−0.05			
**9**	Avoidance craving^a^	0.32	0.08	0.01	−0.05	0.11	0.30	0.24	−0.63**		
**10**	Post-task arousal alcohol condition^b^	−0.01	−0.00	−0.10	−0.09	−0.01	0.07	−0.23	−0.09	−0.12	
**11**	Post-task arousal neutral condition^c^	0.28	0.06	−0.15	−0.04	0.26	0.28	−0.00	0.00	0.19	0.48*

*Notes.* Inhibitory control refers to $d\prime_{\mathrm{adj}}$ scores derived from the Go/NoGo tasks; Working memory capacity refers to parameter K of the working memory task.

*p < .05 **p < .01 ***p < .001, two-tailed.

a Spearman's rank correlations (ρ) were calculated based on a smaller subsample of *n* = 19 (please see *Craving assessment* in the ***Measures*** section).

b Correlations involving post-task arousal in the alcohol condition were calculated controlling for pre-task arousal.

c Correlations involving post-task arousal in the neutral condition were calculated controlling for pre-task arousal.

**Table 4:** Bivariate Spearman rank correlation coefficients (ρ) in the AUD sample (*n* = 24).

		**1**	**2**	**3**	**4**	**5**	**6**	**7**	**8**^a^	**9**^a^	**10**^b^
**1**	Age										
**2**	Education	−0.19									
**3**	Psychotropic medication intake	−0.07	−0.10								
**4**	Factor 2 Psychopathy	−0.12	−0.23	−0.08							
**5**	Inhibitory control alcohol condition	0.13	−0.01	−0.15	−0.58**						
**6**	Inhibitory control neutral condition	0.04	0.02	−0.23	−0.20	0.68***					
**7**	Working memory capacity	0.03	−0.07	−0.06	−0.10	0.16	0.01				
**8**	Approach craving^a^	0.11	−0.58*	−0.10	0.04	−0.23	0.02	−0.28			
**9**	Avoidance craving^a^	0.11	0.18	0.00	−0.14	0.45	0.09	0.08	−0.60*		
**10**	Post-task arousal alcohol condition^b^	−0.29	0.08	−0.36	0.10	−0.02	0.05	0.23	0.27	−0.05	
**11**	Post-task arousal neutral condition^c^	0.03	−0.31	−0.07	−0.07	−0.03	0.38	−0.22	0.33	−0.26	0.16

*Notes*. Inhibitory control refers to $d\prime_{\mathrm{adj}}$ scores derived from the Go/NoGo tasks; Working memory capacity refers to parameter K of the working memory task.

*p < .05 **p < .01 ***p < .001, two-tailed.

a Spearman's rank correlations (ρ) were calculated based on a smaller subsample of *n* = 12 (please see *Craving assessment* in the ***Measures*** section).

b Correlations involving post-task arousal in the alcohol condition were calculated controlling for pre-task arousal.

c Correlations involving post-task arousal in the neutral condition were calculated controlling for pre-task arousal.

### 3.1 Control variables

Regarding age, differential associations with the study variables emerged across the two subsamples. In the SUD sample, age was negatively associated with Factor 2 Psychopathy (ρ = −0.50, *p* = .008) and positively associated with inhibitory control in the neutral condition (ρ = 0.50, *p* = .008). Education was also positively associated with inhibitory control in this group (ρ = 0.42, *p* = .029). No significant correlations with age were observed in the AUD sample, and education showed only isolated associations with cue-reactivity indices. Psychotropic medication use was not significantly related to any of the central study variables in either diagnostic group. Additional exploratory inspections by substance class likewise revealed no systematic associations with inhibitory control, working memory capacity, or Factor 2 scores.

Based on these findings, age and education were included as covariates in subsequent regression models, whereas psychotropic medication intake was not.

### 3.2 Cue-reactivity and inhibitory control

As a preliminary step, associations between cue-reactivity measures (i.e., post-task arousal controlling for baseline, approach and avoidance craving) and inhibitory control were examined.

No significant associations were observed between craving indices and inhibitory control in the alcohol condition in either diagnostic group. Similarly, post-task arousal was not significantly related to inhibitory control across conditions and subsamples (see Table 3, Table 4).

Within the AUD sample, correlations involving craving variables were moderate in magnitude (approach: ρ = −0.23, *p* = .467; avoidance: ρ = 0.45, *p* = .139), but did not reach statistical significance. Given the small subsample size (*n* = 12), however, these patterns should be interpreted with caution and not as reliable evidence of an association.

### 3.3 Inhibitory control and factor 2 psychopathy

#### 3.3.1 Correlational findings

At the bivariate level, negative associations were observed between inhibitory control and PCL:SV Factor 2 across groups and task conditions. These reached statistical significance for inhibitory control in the neutral condition within the SUD sample (ρ = −0.41, *p* = .035) and for inhibitory control in the alcohol-cued condition within the AUD sample (ρ = −0.58, *p* = .003).

#### 3.3.2 Linear regression results

Separate linear regression models were computed for the AUD and SUD samples, including age, education and working memory as covariates, and inhibitory control in the neutral and alcohol-cued conditions as predictors (see Table 5).

**Table 5:** Ordinary least squares regression models in the SUD and AUD samples with inhibitory control in the alcohol-cued and neutral condition predicting Factor 2 Psychopathy alongside control variables.

Outcome: Factor 2 Psychopathy	SUD sample (*n* = 27)					AUD sample (*n* = 24)				
	*F*(5,21) = 2.31, *p* = .080					*F*(5,18) = 3.25, *p* = .029				
	*B*	*SE(B)*	β	*t*	*p*	*B*	*SE(B)*	β	*t*	*p*
(Intercept)	8.05	6.35		1.27	0.219	20.97	4.58		4.58	<0.001
Age	−0.09	0.09	−0.25	−1.03	0.314	−0.04	0.06	−0.11	−0.62	0.544
Education	−0.74	1.01	−0.18	−0.73	0.471	−1.17	0.80	−0.26	−1.47	0.158
Inhibitory control alcohol condition	2.12	1.83	0.30	1.16	0.260	**−5.26**	**1.61**	**−0.79**	**−3.26**	**0.004**
Inhibitory control neutral condition	−2.73	2.05	−0.36	−1.34	0.196	2.06	1.44	0.35	1.43	0.169
Working memory capacity	1.30	0.81	0.33	1.60	0.125	−0.61	0.60	−0.17	−1.01	0.325
										
*R*^*2*^					0.355					0.474
*R*^*2*^_*adj*_					0.202					0.328

*Notes.* Statistically significant associations are highlighted in **bold**. AUD = Alcohol Use Disorder, SUD = Substance Use Disorder, *F* = F-test statistic, *B* = Unstandardized Regression Coeffictient, *SE* = Standard Error, β = Standardized Regression Coefficient, *t* = t-test statistic, *R*^*2*^ = Coefficient of Determination, *R*^*2*^_*adj*_ = Adjusted Coefficient of Determination. Inhibitory control refers to $d\prime_{\mathrm{adj}}$ scores derived from the Go/NoGo tasks; Working memory capacity refers to parameter K of the working memory task.

In the SUD sample, the overall model was not significant, *F*(5,21) = 2.31, *p* = .080, explaining 35.5% of the variance (adjusted *R*^*2*^ = 0.202). None of the predictors reached statistical significance, although inhibitory control in the neutral condition showed the strongest (non-significant) association (β = −0.36, *p* = .196).

In the AUD sample, the model was significant, *F*(5,18) = 3.25, *p* = .029, accounting for 47.4% of the variance in the outcome (adjusted *R*^*2*^ = 0.328). Among the predictors, inhibitory control in the alcohol-cued condition emerged as a statistically significant negative predictor (β = −0.79, *p* = .004), indicating that weaker inhibitory control in response to alcohol-related stimuli was associated with higher Psychopathy Factor 2 scores. All remaining predictor variables were not significant.

## 4 Discussion

The present study examined whether inhibitory control under neutral versus alcohol-cue conditions is associated with criminal recidivism risk in forensic patients committed under Section 64 of the German Criminal Code. By combining an experimental cue-exposure inhibition paradigm with PCL:SV Factor 2 as a validated risk indicator, the study addressed a critical but previously untested question, namely, whether cue-elicited inhibitory impairments as known predictors of substance relapse also relate more directly to criminal behavior risk. The present findings provide initial evidence for such a connection, particularly in patients with AUD.

Across diagnostic groups, descriptive correlations indicated a general tendency for weaker inhibitory control to coincide with higher Factor 2 scores. Crucially, however, this pattern proved diagnosis- and context-specific rather than uniform. In patients with AUD, inhibitory control in the alcohol condition emerged as the central correlate of criminogenic risk. Importantly, this association should be interpreted in terms of individual differences in a stable risk marker rather than as evidence that cue exposure directly alters criminogenic risk. No comparable pattern was observed in the SUD group. This absence of alcohol-specific effects aligns with the idea that alcohol cues carry less motivational salience for individuals whose primary substance of use differs. These group-differentiated patterns underscore the importance of cue-specificity when studying cognitive mechanisms in heterogeneous forensic addiction populations.

The central finding that cue-elicited inhibitory deficits relate to an established proxy of recidivism risk among forensic patients with AUD provides support for the primary hypothesis and extends previous research on cue-reactivity, substance relapse and criminal behavior in several important ways.

First, the findings extend prior research showing that substance cues can impair inhibitory control and promote relapse-related processes (Ames et al., 2014; Field & Jones, 2017; Papachristou et al., 2012, Papachristou et al., 2013). The present results suggest that such cue-dependent impairments may also be relevant for re-engagement in criminal behavior, particularly among forensic patients with AUD. This is consistent with evidence that relapse is a primary pathway to criminal recidivism following forensic addiction treatment (Bezzel, 2010; Passow et al., 2015; Pfaff, 1998). One possible interpretation is that alcohol cues disproportionately disrupt inhibitory control in AUD patients, increasing the likelihood that cue-triggered lapses escalate into criminogenic behavior, particularly in high-risk environments after discharge.

The results also complement theoretical perspectives such as the Impaired Response Inhibition and Salience Attribution (I-RISA) model (Goldstein and Volkow, 2002, Goldstein and Volkow, 2011) and Incentive Sensitization Theory (Robinson and Berridge, 1993, Robinson and Berridge, 2025), which suggest that substance cues can exert strong motivational pull while simultaneously compromising regulatory processes. Notably, the observed association was specific to alcohol-cued inhibition, underscoring the contextual nature of the effect.

The differential pattern between AUD and SUD patients further underscores the role of substance-specific cue salience. Alcohol cues may represent ecologically valid triggers for AUD patients but not for individuals with other primary substances. Accordingly, inhibitory deficits relevant to reoffending risk in the SUD group may emerge under substance-specific cue conditions not captured in the present design.

Thus, the relationship between inhibitory control and criminogenic risk appears to vary as a function of cue context and substance diagnosis. Examining inhibitory control under substance-relevant cue exposure may therefore provide a more nuanced understanding of risk-related mechanisms without implying deterministic conclusions.

Working memory capacity did not significantly contribute to the regression models in either group. This suggests that the observed associations are not merely attributable to broader cognitive deficits but may reflect more specific, contextually triggered impairments.

### 4.1 Limitations

Several limitations warrant consideration when interpreting the present findings. First, sample sizes were modest, particularly in subgroup and craving-related analyses, increasing the risk of Type II error and limiting statistical power and the stability of parameter estimates. The restriction to complete cases further reduced the available sample, potentially affecting representativeness, although missingness was primarily attributable to administrative and technical factors. Second, the present analyses were restricted to male participants, limiting the generalizability of the findings to female forensic populations. These limitations should be interpreted in the context of the structural constraints of research in high-security forensic settings, where recruitment is slow and resource-intensive and often restricted by legal and organizational factors. Replication in larger and more diverse samples will therefore be important, potentially requiring multi-center designs to achieve sufficient statistical power.

Substance specificity is another important limitation of the present work. Because only alcohol-related visual stimuli were used, the task likely failed to capture valid trigger contexts for individuals with primary substances other than alcohol. Consequently, the present conclusions are restricted to alcohol cue-related inhibitory impairments in forensic patients with AUD. Future research should employ substance-tailored cue sets or multimodal cue batteries to more comprehensively assess cue-specific inhibitory impairments across diagnostic groups. In addition, future studies would benefit from incorporating haptic or olfactory cues, virtual reality exposure, simulated purchasing environments, or in vivo exposure scenarios. Such approaches may not only enhance ecological validity but also inform the development of exposure-based interventions or training procedures.

Moreover, the cross-sectional design precludes causal inference. Although the findings are consistent with a risk-relevant role of cue-elicited inhibitory impairments, alternative explanations remain possible. Longitudinal research is needed to determine whether such impairments prospectively predict substance relapse and criminal reoffending.

Several potentially informative moderators and differentiating factors could not be examined due to limited data availability and statistical power. In particular, craving magnitude could not be tested as a moderator of the relationship between inhibitory control and criminogenic risk, as craving data were only available for a reduced subsample and were assessed only after the alcohol-cue condition, precluding the examination of within-session changes. Moreover, cue-reactivity and inhibitory control were not significantly associated in the present data. This pattern is consistent with the possibility that these processes operate in a partially independent or interactive manner. However, given the limited sample size, these findings should be interpreted with caution. The observed effect sizes suggest that potentially meaningful associations may have been present but gone undetected due to limited statistical power. Future research should therefore examine these relationships in larger samples and explicitly test interaction models to better capture the combined influence of bottom-up and top-down processes.

Notably, no robust group differences in inhibitory performance emerged at the descriptive level, with diagnostic specificity emerging primarily in the pattern of associations between inhibitory control and PCL:SV Factor 2. This suggests that inhibitory deficits may be more relevant in their context-dependent relationship with criminogenic risk rather than as global impairments. In addition, task characteristics may have influenced the findings, as different paradigms (e.g., Stop-Signal tasks) capture distinct aspects of inhibitory control (Littman & Takács, 2017; Verbruggen & Logan, 2008).

Finally, all participants were admitted under the pre-reform version of Section 64 of the German Criminal Code. Changes introduced in the 2023 reform may affect the composition of future samples of forensic patients and, consequently, the generalizability of the present findings. Given the stricter causality and prognostic criteria, it is plausible that associations between substance-related cognitive mechanisms and criminogenic risk may be more pronounced in post-reform populations (Schwarz & Stübner, 2023; Soyka, 2024). However, this remains an open empirical question that requires direct investigation once sufficient post-reform data become available.

### 4.2 Implications

Despite these limitations, the present findings carry several important implications for future forensic research and clinical practice. First, they suggest that assessing inhibitory control under substance-relevant cue exposure may provide incremental value to forensic risk assessment, particularly in individuals with AUD. In this study, inhibitory performance under alcohol-cued conditions, but not under neutral conditions, was associated with a validated proxy of recidivism risk, suggesting the importance of context-sensitive cognitive assessment. Such paradigms may help capture risk-relevant dysregulation that remains undetected in neutral neuropsychological tasks or global indices of impulse control, and may thus complement established structured risk assessments with objective measures.

Second, the findings have implications for the development and refinement of treatment approaches in forensic addiction settings. A growing body of evidence suggests that inhibitory control can be modified through computerized inhibitory control training (ICT), particularly when paradigms are cue-specific and employ Go/NoGo tasks: Meta-analytic work suggests that such training produces small but relatively consistent short-term effects on behavior, improves inhibitory control, and reduces the motivational value of substance cues, especially when objective outcome measures are used (Allom et al., 2016; Iannazzo et al., 2025; Jones et al., 2016). Within a forensic context, such interventions could be implemented within secure treatment settings as a structured cognitive training component aimed at strengthening regulatory capacities in the presence of substance cues.

Complementary meta-analytic evidence from cue exposure therapy (CET) indicates that exposure-based interventions are associated with small to moderate benefits among individuals with AUD, particularly regarding drinking intensity, drinking frequency, and relapse-related outcomes, although effect sizes are heterogeneous and the overall quality of evidence remains low (Kiyak et al., 2023; Mellentin et al., 2017). Both meta-analyses suggest that CET is more effective when combined with craving-related coping skills training practiced directly in the presence of substance cues, as compared to conventional CET alone. Accordingly, extinction processes may be strengthened when cue exposure is paired with active regulatory strategies rather than passive exposure alone. From this perspective, ICT may serve as a foundational intervention that strengthens inhibitory control and attenuates automatic cue-driven responding, while CET, particularly when augmented by coping skills training, may constitute a second layer that supports the application of these regulatory capacities under motivationally salient conditions.

The secure and structured nature of forensic inpatient settings offers a unique opportunity to examine and refine such integrated approaches. Unlike community-based samples, patients in forensic addiction treatment are abstinent over extended periods, exposure to cues can be systematically controlled, and training effects can be monitored without the confounding influence of ongoing substance use. Therefore, cognitive training effects may be consolidated before transfer to real-world cue exposure contexts. At the same time, it remains an open empirical question whether substance-related stimuli retain the same appetitive value in custodial settings as they do in naturalistic environments, or whether their motivational salience changes over the course of treatment. This distinction is critical, as it may influence whether interventions primarily target appetitive extinction, regulatory skill application, or both.

Taken together, if replicated and extended longitudinally, cue-based assessments of inhibitory control may help identify tailored recidivism risk assessment and intervention strategies in forensic addiction treatment. At the same time, the present implications remain tentative and underscore the need for larger, post-reform samples and prospective designs that incorporate actual substance relapse and criminal reoffending outcomes before such approaches can be translated into routine forensic practice.

## 5 Conclusion and outlook

The present work provides seminal evidence that inhibitory control deficits elicited by alcohol cues are associated with an established proxy of criminal recidivism risk among forensic patients with AUD, but not among those with other SUDs. These results highlight the context- and diagnosis-specific nature of inhibitory impairments and their potential relevance for understanding the pathways linking substance use, criminal behavior and reoffending. Integrating cue-specific cognitive assessments into forensic practice may therefore contribute to more nuanced risk evaluation and inform future approaches to targeted intervention strategies aimed at reducing relapse and reoffending.

## Declaration of generative AI and AI-assisted technologies in the manuscript preparation process

During the preparation of this work the authors utilized DeepL Write (https://www.deepl.com/write/) and ChatGPT-5.2 (https://chat.openai.com/) in order to improve language clarity and prevent grammatical errors. After using these services, the authors carefully reviewed and edited the content as needed. The authors take full responsibility for the final content of this publication.

## CRediT authorship contribution statement

**Helena Sophia Schmitt:** Writing – review & editing, Writing – original draft, Visualization, Software, Project administration, Methodology, Investigation, Formal analysis, Data curation, Conceptualization. **Jennifer Wernicke:** Writing – review & editing, Software, Methodology, Formal analysis. **Cornelia Sindermann:** Writing – review & editing. **Ralf Wilhelm Wolf:** Writing – review & editing, Resources, Project administration, Conceptualization. **Sebastian Markett:** Writing – review & editing, Software, Resources. **Esther Ott:** Writing – review & editing, Validation, Formal analysis. **Christian Montag:** Writing – review & editing, Supervision, Project administration, Conceptualization.

## Funding sources

This work was supported by resources available to Prof. Dr. Christian Montag at Ulm University. Helena Sophia Schmitt was a doctoral scholarship holder at the German Academic Scholarship Foundation (Studienstiftung des Deutschen Volkes) [Doctoral scholarship].

## Declaration of competing interest

The authors declare that they have no known competing financial interests or personal relationships that could have appeared to influence the work reported in this paper.

## Data Availability

The data that has been used is confidential.
