# Supplementary material for: Cue-induced inhibitory control in forensic patients with alcohol use disorder: A link to criminal recidivism risk assessed by factor 2 psychopathy
Source: Addict Behav Rep. 2026 May 27;24:100713. doi: 10.1016/j.abrep.2026.100713 (PMC13315866; doi:10.1016/j.abrep.2026.100713)
Supplement: Supplementary file 1 — Supplementary material [file mmc1.pdf]

Cue-induced inhibitory control in forensic patients with alcohol use disorder: A link to criminal recidivism risk assessed by factor 2 psychopathy

Supplementary Materials

Supplementary Materials S1. Go/NoGo Task Details and Preprocessing Steps.

Task parameters were selected to reliably elicit a prepotent response tendency and sufficient inhibitory demand. A Go:NoGo ratio of 3:1 (75%/25%) and relatively fast trial timing were implemented in line with methodological evidence indicating that rare NoGo events and faster pacing enhance inhibitory engagement compared to equiprobable or slow-paced designs (Wang et al., 2025; Wessel, 2018; Young et al., 2018). Stimulus presentation duration followed a prior alcohol-related inhibitory control paradigm (Kreusch et al., 2014). All stimuli were presented in the center of a white background. Each condition started with general written instructions.

During an exercise block consisting of 28 trials with the letters “X” as Go stimulus and “O” as NoGo stimulus, the study coordinator monitored the exercise to ensure correct understanding and performance. Afterwards, the actual experimental testing began. The experiment consisted of six blocks comprising 80 trials each. Thus, in each block, 60 trials presented Go stimuli requiring a spacebar press, while 20 trials presented NoGo stimuli for which participants were required to withhold their responses. In the first four blocks (Blocks 1-4), one Go stimulus and one NoGo stimulus were used. In the final two blocks (Blocks 5-6), task complexity was increased by presenting two different Go stimuli (30 trials each) and one NoGo stimulus. The sequence of stimuli within each block was randomized.

The temporal structure of the experiment is illustrated in **Figure 2** of the main article. In the alcohol cue-exposure condition, colored images of beverages were shown, following prior alcohol Go/NoGo paradigms (Ames et al., 2014; Kreusch et al., 2014). Across blocks, both open drinks served in containers and beverage brand labels were presented. Go stimuli always depicted non-alcoholic beverages (e.g., water, coffee, cola),

whereas NoGo stimuli depicted alcoholic beverages (e.g., beer, vodka, whiskey). In the neutral condition, neutral, non-affective stimuli consisting of simple two-dimensional geometric shapes (e.g., square, triangle, circle) were presented (Benikos et al., 2013).

For each participant and each block, the number of correct responses in Go trials (hits) and incorrect responses in NoGo trials (false alarms) were determined. To rule out random hits, responses were only counted as hits if their corresponding reaction times (RTs) fell within an individual confidence interval ($M \pm$ 2 SD of the participant's own hit RTs) for each participant. Responses outside this range were not counted as hits.

Task performance was quantified using the sensitivity parameter d' from signal detection theory, which reflects the difference between the z-transformed hit rate and the z-transformed false alarm rate (Macmillan, 2002). Because extreme proportions (i.e., a hit rate of 1 or a false-alarm rate of 0) yield infinite values, the log-linear correction proposed by Hautus (1995) was applied. In this procedure, 0.5 is added to each cell of the 2x2 contingency table, thereby increasing the denominators of the proportions by +1.

The adjusted proportions were thus calculated as follows:

$$H_{adj} = \frac{Hits + 0.5}{n_{\mathrm{Go}} + 1} = \frac{Hits + 0.5}{61}$$

$$F_{adj} = \frac{False\ Alarms + 0.5}{n_{\mathrm{NoGo}} + 1} = \frac{False\ Alarms + 0.5}{21}$$

The adjusted sensitivity metric reported in this study was therefore calculated as:

$$d'_{adj} = \mathrm{Z}(H_{adj}) - \mathrm{Z}(F_{adj})$$

where $Z(x)$ denotes the inverse normal distribution, H_{adj} the adjusted hit rate, and F_{adj} the adjusted false alarm rate.

The adjusted d' was calculated separately for each block. Task performance was inspected at the block level to identify implausible or noncompliant response patterns

across the course of the task. Two participants showed negative d'_{adj} values across multiple blocks in the neutral condition, indicating indiscriminate responding or failure to follow task instructions (Young et al., 2018), and were therefore excluded from further analyses. Subsequently, mean d'_{adj} values were computed across the six blocks for the neutral and alcohol conditions.

Supplementary Materials S2. Working Memory Task Details and Preprocessing Steps.

Working memory capacity was assessed using a change-detection task following the procedure of Luck and Vogel (1997) and as described in Markett et al. (2018). In addition to written instructions describing the paradigm, participants were shown an illustrative example trial. This was followed by an exercise run in which participants received on-screen feedback regarding the accuracy of their responses. The actual test phase began immediately afterwards. Participants completed four blocks of 32 trials each.

Each trial began with a centrally presented fixation cross (500 ms), followed by a stimulus array consisting of colored squares. The squares were displayed in black, blue, green, purple, red, white, or yellow, with no color repeated within a single trial. All stimuli were shown on a gray background. The number of squares increased by one across blocks, starting with three squares in Block 1 and ending with six squares in Block 4. After a stimulus-free retention interval (1000 ms), a probe array appeared. The probe array was either identical to the original array (catch trial) or differed in the color of exactly one square (change trial). An example trial is shown in **Figure 3** of the main article. The probe array remained on the screen until participants responded. Each trial type occurred 16 times per block, and participants were instructed to indicate as quickly as possible whether all squares had remained the same color (press "j") or whether one square had changed color (press "f"). A 1000 ms ITI was used between trials.

Individual working memory capacity was calculated for each block using the Pashler formula (Pashler, 1988):

$$K = \frac{HR - FR}{1 - FR} \times N$$

where HR represents the hit rate on change trials, FR the false alarm rate on catch trials, and N the number of squares presented in the respective block. As the measure of

individual working memory performance, the highest K value obtained by a given participant across the four blocks was used, as this value most reliably reflects the individual's capacity limit.

Supplementary Materials S3. Regression diagnostics

To test the core hypothesis of the present work, linear regressions were conducted within groups. Regression assumptions were tested by entering the predictors of interest, i.e., inhibitory control in the alcohol and neutral conditions alongside working memory capacity into regression models with PCL:SV Factor 2 scores as the outcomes. The assumption of linearity between predictors and the outcome, as well as the homoscedasticity of residuals, was checked visually using scatterplots and found to be met. The independence of residuals was confirmed using the Durbin-Watson (DW) statistic ($DW_{AUD} = 2.10$; $DW_{SUD} = 2.08$). Tolerance and variance inflation factor (VIF) values indicated absence of multicollinearity ($.504 \leq Tolerance_{AUD} \leq .992$, $1.008 \leq VIF_{AUD} \leq 1.983$; $.603 \leq Tolerance_{SUD} \leq .933$, $1.072 \leq VIF_{SUD} \leq 1.658$). Normality of residuals was confirmed using the Shapiro-Wilk test ($W_{AUD} = .965$, $p = .538$; $W_{SUD} = .956$, $p = .306$).
To detect potential outlier cases in regression analyses, Cook's *D*, standardized differences in fit (*DFFITS*), and standardized differences in beta (*DFBETAS*) were analyzed, following Aguinis et al. (2013). One case per diagnostic group exceeded the respective cutoffs and was classified as a potential prediction outlier. Sensitivity analyses showed that excluding these cases led to somewhat stronger but overall comparable results. Given the small sample sizes and the winsorization already applied, both cases were retained in the main analyses to preserve variance. Based on the assessment of regression assumptions, ordinary least squares (OLS) regressions were conducted in both groups.

References

Aguinis, H., Gottfredson, R. K., & Joo, H. (2013). Best-Practice Recommendations for Defining, Identifying, and Handling Outliers. *Organizational Research Methods*, *16*(2), 270–301. https://doi.org/10.1177/1094428112470848

Ames, S. L., Wong, S. W., Bechara, A., Cappelli, C., Dust, M., Grenard, J. L., & Stacy, A. W. (2014). Neural correlates of a Go/NoGo task with alcohol stimuli in light and heavy young drinkers. *Behavioural Brain Research*, *274*, 382–389. https://doi.org/10.1016/j.bbr.2014.08.039

Benikos, N., Johnstone, S. J., & Roodenrys, S. J. (2013). Short-term training in the Go/Nogo task: Behavioural and neural changes depend on task demands. *International Journal of Psychophysiology*, *87*(3), 301–312. https://doi.org/10.1016/j.ijpsycho.2012.12.001

Hautus, M. J. (1995). Corrections for extreme proportions and their biasing effects on estimated values ofd′. *Behavior Research Methods, Instruments, & Computers*, *27*(1), 46–51. https://doi.org/10.3758/BF03203619

Kreusch, F., Quertemont, E., Vilenne, A., & Hansenne, M. (2014). Alcohol abuse and ERP components in Go/No-go tasks using alcohol-related stimuli: Impact of alcohol avoidance. *International Journal of Psychophysiology*, *94*(1), 92–99. https://doi.org/10.1016/j.ijpsycho.2014.08.001

Luck, S. J., & Vogel, E. K. (1997). The capacity of visual working memory for features and conjunctions. *Nature*, *390*(6657), 279–281. https://doi.org/10.1038/36846

Macmillan, N. A. (2002). Signal detection theory. In *Stevens' handbook of experimental psychology: Methodology in experimental psychology* (3rd ed., Vol. 4, pp. 43–90). John Wiley & Sons, Inc.

Markett, S., Reuter, M., Heeren, B., Lachmann, B., Weber, B., & Montag, C. (2018). Working memory capacity and the functional connectome—Insights from resting-state fMRI and voxelwise centrality mapping. *Brain Imaging and Behavior*, *12*(1), 238–246. https://doi.org/10.1007/s11682-017-9688-9

Pashler, H. (1988). Familiarity and visual change detection. *Perception & Psychophysics*, *44*(4), 369–378. https://doi.org/10.3758/BF03210419

Wang, X., Ren, G., Gao, H., & Qi, M. (2025). The effectiveness of Go/Nogo dual-task is modulated by SOA and Go/Nogo ratio. *Behavioural Processes*, *231*, 105255. https://doi.org/10.1016/j.beproc.2025.105255

Wessel, J. R. (2018). Prepotent motor activity and inhibitory control demands in different variants of the go/no-go paradigm. *Psychophysiology*, *55*(3), e12871. https://doi.org/10.1111/psyp.12871

Young, M. E., Sutherland, S. C., & McCoy, A. W. (2018). Optimal go/no-go ratios to maximize false alarms. *Behavior Research Methods*, *50*(3), 1020–1029. https://doi.org/10.3758/s13428-017-0923-5
